# Characterization of intrahepatic cholangiocarcinoma after curative resection: outcome, prognostic factor, and recurrence

**DOI:** 10.1186/s12876-018-0912-x

**Published:** 2018-12-04

**Authors:** Kun-Ming Chan, Chun-Yi Tsai, Chun-Nan Yeh, Ta-Sen Yeh, Wei-Chen Lee, Yi-Yin Jan, Miin-Fu Chen

**Affiliations:** Department of General Surgery, Chang Gung Memorial Hospital at Linkou, Chang Gung University College of Medicine, 5 Fu-Hsing Street, Kwei-Shan District, Taoyuan City, 33305 Taiwan

**Keywords:** Intrahepatic cholangiocarcinoma, Curative resection, Recurrence, Prognostic factors, Outcome

## Abstract

**Background:**

Intrahepatic cholangiocarcinoma (ICC) is a relatively rare subtype of cholangiocarcinoma. The study herein gathered experience of surgical treatment for ICC, and aimed to analyze the prognosis of patients who had received curative-intent liver resection.

**Methods:**

A total of 216 patients who had undergone curative-intent liver resection for ICC between January 1977 and December 2014 was retrospectively reviewed.

**Results:**

Overall, the rates of 5-years recurrence-free survival (RFS) and overall survival (OS) were 26.1 and 33.9% respectively. Based on multivariate analysis, four independent adverse prognostic factors including morphology patterns, maximum tumor size > 5 cm, pathological lymph node involvement, and vascular invasion were identified as affecting RFS after curative-intent liver resection for ICC. Among patients with cholangiocarcinoma recurrence, only 27 (16.9%) were able to receive surgical resection for recurrent cholangiocarcinoma that had a significantly better outcome than the remaining patients.

**Conclusion:**

Despite curative resection, the general outcome of patients with ICC is still unsatisfactory because of a high incidence of cholangiocarcinoma recurrence after operation. Tumor factors associated with cholangiocarcinoma remain crucial for the prognosis of patients with ICC after curative liver resection. Moreover, aggressive attitude toward repeat resection for the postoperative recurrent cholangiocarcinoma could provide a favorable outcome for patients.

## Background

Intrahepatic cholangiocarcinoma (ICC) is a primary liver malignancy arising from the epithelial cells of the distal branch intrahepatic bile duct. The incidence of ICC exhibits wide geographical variation and generally accounts for between 5 and 30% of primary liver cancers [1–4]. There has been a noticeable increase in the incidence of ICC in Western countries in recent years [5].

Currently, surgical resection with curative intent remains the most effective treatment for ICC. However, because of vague symptomatic presentation, most patients are at an advanced stage by the time of diagnosis, and only nearly one-third of patients are eligible for surgical resection [6]. As a result, the overall outcome of ICC remains extremely poor, in which patients who are unable to undergo surgical resection have a less than 10% survival rate at 5 years. Moreover, the reported outcome after hepatic resection is also not optimistic, with a 5-year survival rate of 30 to 35% [7]. The principal reason for the dismal outcome of surgical treatment is the high incidence of postoperative ICC recurrence, in which more than 60% of patients may subsequently develop cancer recurrence after hepatic resection.

As a noteworthy malignancy, predictors for ICC recurrence and long-term outcome following hepatic resection remains entirely elusive. In addition, this remains an issue of great concern despite a growing experience and literature. Therefore, here we retrospectively reviewed our experience with surgical resection for ICC patients with the aim of providing additional information about the prognostic factors associated with those patients undergoing curative-intent liver resection, as well as the outcomes of ICC recurrence after surgical treatment.

## Materials and methods

### Patients

This study included patients with ICC who underwent surgical treatment with curative resection between January 1977 and December 2014 at Chang Gung Memorial Hospital, Linkou Medical Center, Taoyuan, Taiwan. A retrospective review of all medical records was performed under the approval of the Institutional Review Board of Chang Gung Memorial Hospital (Approval No.: 201701127B0). The medical records, including clinical characteristics, surgical management, and outcomes were thoroughly reviewed and analyzed. Patients who had no curative resection with macroscopically and/or microscopically positive of carcinoma at the resection margin were not included in the study. Therefore, a total of 225 patients who had pathological confirmation of cholangiocarcinoma were retrieved. After exclusion of 9 patients (4%) with postoperative hospital mortality, 216 patients [99 men (45.8%) and 117 women (54.2%)] were recruited and analyzed for this study.

### Liver resection and follow-up

Transection of hepatic parenchymal was performed using either the surgical clamp-crush technique or a Cavitron Ultrasonic Surgical Aspirator (CUSA; Valleylab, Inc., Integra LifeSciences, Plainsboro, NJ). However, liver resection was mostly performed by CUSA transection after it was introduced into our institute in 2002. After the operation, all patients were followed-up at regular intervals until death or the end of the current study. The clinical assessments included physical examination, blood chemistry tests, measurement of tumor-markers, and abdominal ultrasonography every 3–6 months. A comprehensive assessment was done using computed tomography (CT) and/or magnetic resonance imaging (MRI) on an annual basis or when suspicious of cancer recurrence.

Based on the pathological examination, cancer was staged according to the 7th edition of tumor-node-metastasis (TNM) classification proposed by the Union for International Cancer Control (UICC) and the American Joint Committee on Cancer (AJCC) to classify the extent of cholangiocarcinoma. Patients who had cancer staged by former version of classification system were restaged by the 7th edition of UICC/AJCC classification. The administration of postoperative adjuvant chemotherapy was optional and mainly based on tumor characteristics, patient’s physical condition, and availability or affordability of chemotherapeutic regimens. The chemotherapeutic options were mostly fluorouracil plus leucovorin and/or a combination of regimens such as cisplatin, mitomycin, oxaliplatin, gemcitabine, and so on.

Disease recurrence was determined by a tissue sample from either a biopsy or surgical resection confirming cholangiocarcinoma, and/or by serial imaging examinations. Generally, the treatment algorithm of recurrent cholangiocarcinoma after surgical resection was the same as that for the initial management of cholangiocarcinoma. Repeat surgical resection was the preferred treatment whenever the recurrent tumor was considered to be resectable. Palliative chemotherapy was usually recommended for patients who had unresectable recurrent tumor or not received reoperation unless a patient was unsuitable for chemotherapy or unwilling to receive chemotherapy.

### Outcome and statistical analysis

The end-point outcome measures included recurrence-free survival (RFS) and overall survival (OS). RFS was defined as the date of liver resection to the date of detected cholangiocarcinoma recurrence or the date of the last follow-up if there was no cancer recurrence. OS was measured from the date of liver resection to the date of death or the date of the last follow-up by the end of this study. Survival curves were constructed using the Kaplan–Meier method and analyzed by means of the log-rank test. The categorical variables were assessed using the χ^2^ or Fisher exact test as appropriate, and the independent samples *t*-test was used for continuous data. Variables were analyzed using a Cox regression proportional hazards model to identify factors influencing RFS and OS. All significant factors determined by univariate analysis were then entered into a multivariate analysis using the Cox proportional hazards regression model. All statistical analyses were performed using SPSS statistical software version 20.0 (SPSS, Inc., Chicago, IL) for Windows. A *P*-value of less than 0.05 was considered to be statistically significant.

## Results

### Clinical features of patients

Table 1 summarizes the clinical features of the 216 patients who underwent curative-intent liver resection for ICC in this study. The median age of patients at the time of initial diagnoses was 60-years-old and ranged from 29 to 90-years-old. The majority of patients (90.3%) were not associated with liver cirrhosis, and 23.2% of patients were noted as having the simultaneous presence of hepatolithiasis in the biliary tree. Of these, major hepatectomy (≥ 3 hepatic segments according to Couinaud’s definition) was performed for 126 patients (58.3%), and the remaining 90 patients (41.7%) underwent minor hepatectomy (< 3 hepatic segments). Meanwhile, 11 patients (5.1%) underwent simultaneous bile duct resection. The majority of patients (50.9%) underwent liver resection during the last decade of the study period.

**Table 1:** Clinical characteristics of patients undergoing curative resection for intrahepatic cholangiocarcinoma

Characteristics	Patients *n* = 216(%)
Age (years), median (range)	60.0 (29–90)
Gender	
Male	99 (45.8)
Female	117 (54.2)
Liver cirrhosis	
Yes	21 (9.7)
No	195 (90.3)
Hepatolithiasis	
Yes	51 (23.2)
No	165 (76.8)
Virus hepatitis	
HBV positive	48 (22.2)
HCV positive	19 (8.8)
Extent of hepatic resection	
≥ 3 segments	126 (58.3)
< 3 segments	90 (41.7)
Extrahepatic bile duct resection	11 (5.1)
Years of liver resection	
1977–1994	31 (14.4)
1995–2004	75 (34.7)
2005–2014	110 (50.9)
Morphology type	
Intraductal growth	42 (19.4)
Mass-forming	123 (56.9)
Mix type	21 (9.7)
Periductal-infiltrating	30 (13.9)
TNM stage	
I	103 (47.7)
II	18 (8.3)
III	24 (11.1)
IVA	71 (32.9)

*HBV* Hepatitis B virus, *HCV* Hepatitis C virus

### Patient’s outcome

**Fig. 1:**
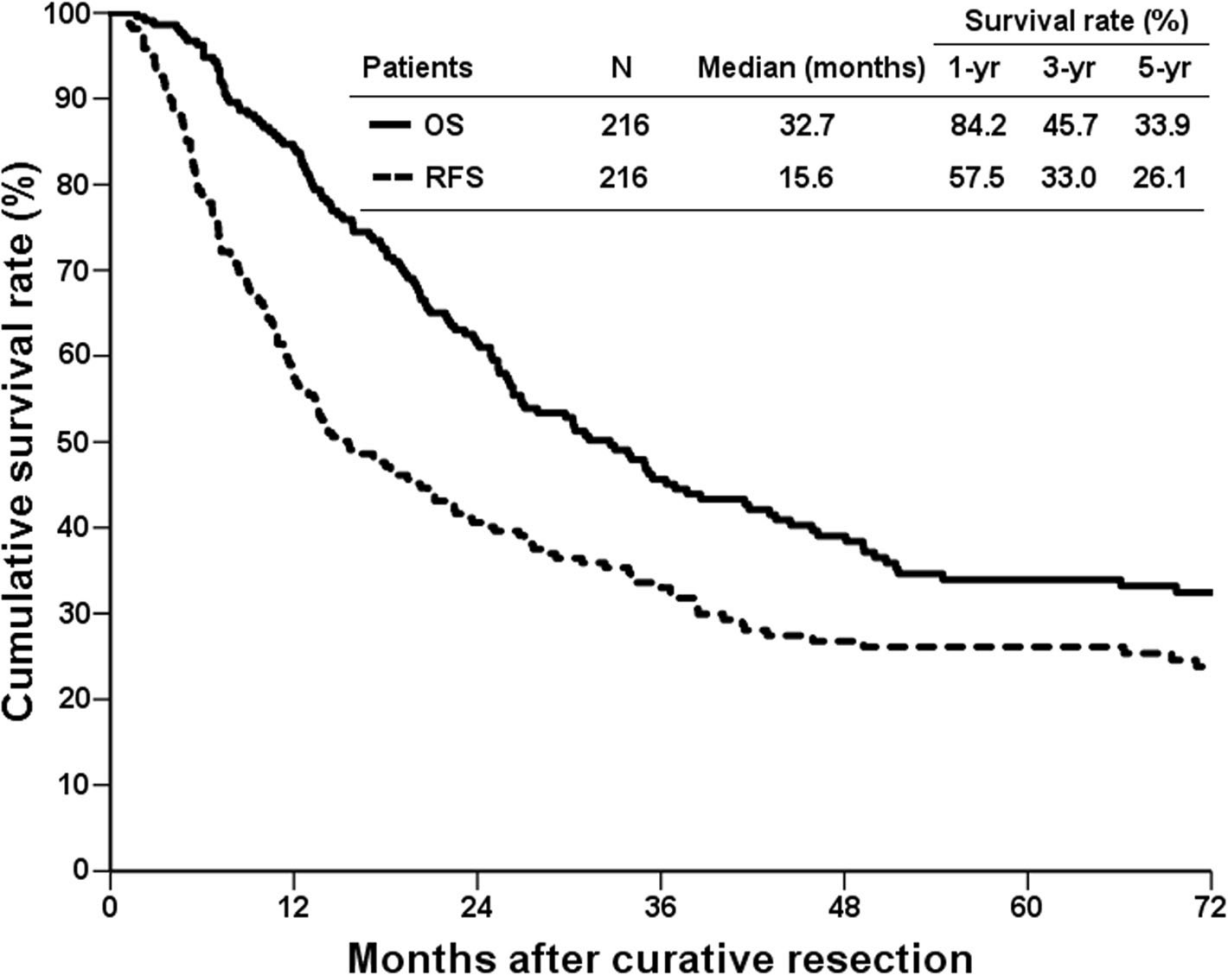
Kaplan-Meier cumulative survival curves of the patients who underwent curative resection for intrahepatic cholangiocarcinoma by recurrence-free survival (RFS) and overall survival (OS)

The median follow-up time for all patients was 26.9 months (range, 1.7 to 268). Overall, 160 patients (74.1%) encountered cancer recurrence after liver resection, and 56 (25.9%) patients had no cancer recurrence by the date of last follow-up or the end of this study. Meanwhile, 168 (77.8%) patients had died during the follow-up period, in which 145 (67.1%) patients died of cholangiocarcinoma, and 23 (10.6%) patients died of diseases other than cholangiocarcinoma. Only 42 (19.4%) patients were still alive by the end of the study, including 28 (13.0%) patients who were cancer free and 14 (6.5%) patients alive with recurrent cholangiocarcinoma. The remaining 6 (2.8%) patients were lost during the follow-up period. The RFS and OS curves are shown in Fig. 1. The 1-, 3-, and 5-year RFS rates were 57.5, 33.0, and 26.1% respectively, and the 1-, 3-, and 5-year OS rates were 84.2, 45.7, and 33.9% respectively.

### Prognostic factors affecting cancer recurrence

The prognostic factors affecting cholangiocarcinoma recurrence after curative-intent liver resection were analyzed and summarized in Table 2. Univariate analysis identified nine significant factors including morphology patterns, histologic differentiation, maximum tumor size, pathological T stage, pathological N stage, vascular invasion, perineural invasion, and postoperative adjuvant chemotherapy. Subsequently, multivariate regression analysis of these significant factors showed that morphology patterns, maximum tumor size > 5 cm, pathological lymph node involvement, and vascular invasion were independent risk factors for cholangiocarcinoma recurrence after liver resection.

**Table 2:** Univariate and multivariate analyses of clinicopathological factors affecting RFS after curative resections of patients with ICC

Factors	Univariate analysis			Multivariate analysis	
	medium RFS months	95%CI	*p* value	HR(95%CI)	*p* value
Age (years)					
≤ 65	13.5	8.6–18.5	0.320	–	
> 65	18.3	11.0–25.7			
Gender					
Male	18.0	10.4–25.5	0.962	–	
Female	13.8	8.8–18.8			
Liver cirrhosis					
Yes	14.0	3.6–24.5	0.963	–	
No	15.6	10.5–20.8			
Hepatolithiasis					
Yes	21.0	5.4–36.7	0.856	–	
No	14.2	9.8–18.7			
Years of liver resection					
1977–1994	38.4	12.7–64.2	0.688	–	
1995–2004	13.0	10.3–15.6			
2005–2014	15.7	1.6–20.7			
Morphology patterns					
Intraductal growth	71.0	6.7–135.3	< 0.0001	1	
Mass-forming	7.0	4.8–9.3		1.87 (1.11–3.13)	< 0.001
Mix type	16.4	10.5–20.3		2.59 (1.13–5.95)	0.018
Periductal-infiltrating	10.3	2.2–18.3		4.43 (2.09–9.38)	0.025
Histologic differentiation					
Well, moderate	19.6	11.9–27.3	0.004	1	
Poor, undifferentiated	10.1	6.7–13.6		1.13 (0.78–1.62)	0.522
Maximum tumor size					
≤ 5 cm	25.1	13.0–37.2	< 0.0001	1	
> 5 cm	10.7	8.2–13.3		1.52 (1.07–2.15)	0.019
Pathological T stage					
T1–2	32.4	19.6–45.1	< 0.0001	1	
T3–4	8.0	5.4–10.6		1.02 (0.63–1.66)	0.931
Pathological N stage					
N0	22.5	15.4–29.6	< 0.0001	1	
N1	6.1	4.3–8.0		2.67 (1.59–4.48)	< 0.001
Vascular invasion					
No	20.3	14.3–26.2	< 0.0001	1	
Yes	6.9	3.7–10.0		2.43 (1.54–3.84)	< 0.001
Perineural invasion					
No	22.3	15.0–29.6	< 0.0001	1	
Yes	10.3	6.0–14.6		1.02 (0.65–1.62)	0.921
Adjuvant chemotherapy					
No	20.3	14.0–26.6	0.023	1	0.517
Yes	12.5	10.7–14.3		0.89 (0.62–1.26)	

*ICC* Intrahepatic cholangiocarcinoma, *RFS* Recurrence-free survival, *HR* Hazard ratio, *CI* Confidence interval

### Recurrence after curative-intent liver resection

**Fig. 2:**
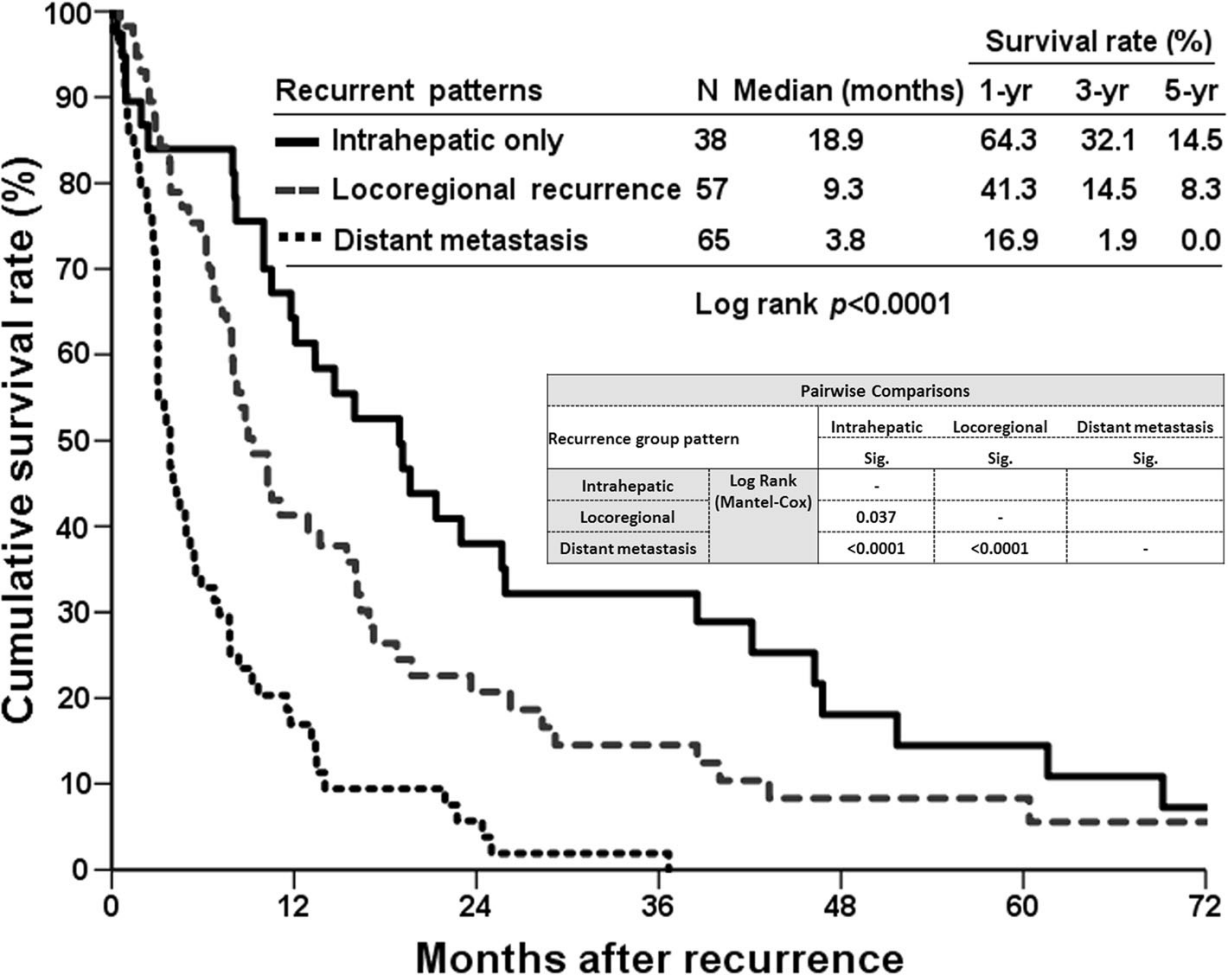
Among patients with postoperative recurrence, the survival curves are compared according to recurrent patterns. Patients with only intrahepatic recurrence had a significantly better survival curve than other two recurrent patterns (*p* < 0.0001)

**Fig. 3:**
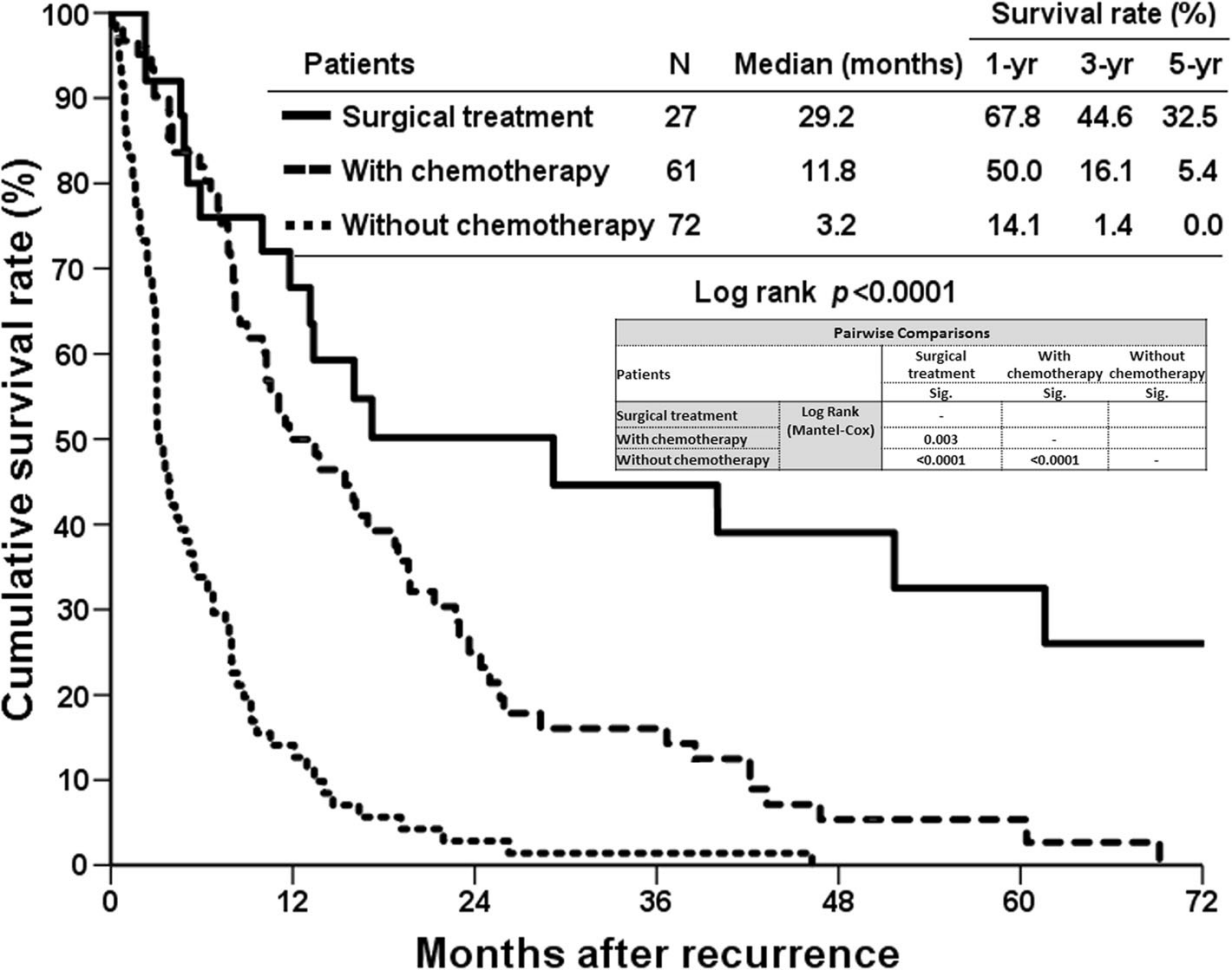
Kaplan-Meier survival curves of the patients with cholangiocarcinoma recurrence after curative resection. The patients who underwent surgical resection for recurrent cholangiocarcinoma had a significantly better survival curve than those who did not undergo surgical resection for recurrent cholangiocarcinoma. Among patients without surgical treatment for recurrent cholangiocarcinoma, the survival curve of patients who had received palliative chemotherapy was better than that of patients without palliative chemotherapy (*p* < 0.0001)

Of the 160 patients who developed cancer recurrence after curative-intent liver resection, 38 (23.8%) patients occurred only at the intrahepatic area, 57 (35.6%) patients had locoregional recurrence with (*n* = 22) or without (*n* = 35) intrahepatic recurrence, and 65 (40.6%) patients had distant metastasis at the detection of cancer recurrence. Table 3 summarizes the location of cholangiocarcinoma recurrence. Only 27 (16.9%) patients were able to receive surgical resection for recurrent lesions. The overall survival based on recurrent patterns showed that patients with intrahepatic recurrence had better survival than those with the other recurrence types. The 5-year survival rates were 14.5, 8.3, and 0% for intrahepatic recurrence, locoregional recurrence, and distant metastasis respectively (Fig. 2). The survival curve of patients who had undergone repeat surgical resection for recurrent cholangiocarcinoma was better than that of patients who were unable to undergo surgical resection, in which the 5-year survival rates after cholangiocarcinoma recurrence were 32.5%. With regard to patients without surgical treatment for recurrent cholangiocarcinoma, the survival curve of patients who had received palliative chemotherapy was better than that of patients without palliative chemotherapy. The 5-year survival rate of patients with palliative chemotherapy was 5.4%, and patients without palliative chemotherapy could not survive more than 5 years reflected by 0% of 5-year survival rate (Fig. 3).

**Table 3:** Surgical resection of recurrent lesions based on the recurrent patterns

Recurrent features	Recurrence^a^	Surgical resection^b^
Number of patients	160	27 (16.9%)
Recurrent patterns		
Intrahepatic only	38 (23.8%)	12 (57.9%)
Locoregional		
with intrahepatic lesion	22 (13.8%)	2 (9.1%)
without intrahepatic lesion	35 (21.9%)	9 (25.7%)
Distant metastasis	65 (40.6%)	4 (6.2%)

^a^percentages represent the ratio among total recurrences; ^b^percentages represent the ratio among recurrent cases

## Discussion

Cholangiocarcinoma is the second most common primary liver cancer following hepatocellular carcinoma despite being rare in clinical practice, and generally accounting for 10–15% of primary hepatic malignancy. ICC is a relatively rare subtype and represents less than 10% of cholangiocarcinoma cases [2, 8]. Although surgical resection is undoubtedly the most effective treatment for ICC, its low resectability and high incidence of postoperative recurrence affect the overall outcome of patients with ICC. Here we gathered data from decades of treating ICC and show that the rate of long-term cancer recurrence remains high (up to 74%). Meanwhile, prognostic factors affecting cancer recurrence after curative resection and outcome of patients after recurrent disease were also elucidated, providing further understanding in terms of the therapeutic strategies of ICC.

Cholangiocarcinoma usually arises from epithelial cells of the biliary tract and could be distinguished by anatomic location and classified as intrahepatic, perihilar, or extrahepatic types. Additionally, outcomes based on these classifications are also varied in a clinical setting. Among the three types, ICC accounts for less than 10% of all cholangiocarcinoma but seems to have the best outcome of the three types [8]. Currently, no specific risk factors are identified association with cholangiocarcinoma, and most cancer arises de novo. Although numerous studies have recognized that cirrhosis, viral hepatitis B and C, primary sclerosing cholangitis, and hepatolithiasis could be risk factors for cholangiocarcinoma, data reported from eastern and western countries are not identical [9–14]. Therefore, there is a lack of consensus on the guideline of risk stratification for disease surveillance.

Additionally, the high incidence of disease recurrence after surgical resection remains a major concern. Numerous studies have reported several prognostic factors that affect the outcomes of patients who undergo surgical resection for ICC [15–18], and similar factors were also noted in this study. The size of the primary tumor and presence of lymph node involvement seem to be important risk factors for cholangiocarcinoma recurrence after surgical resection. Although the 7th edition of UICC/AJCC TNM staging system does not mention tumor diameter, tumor diameter remains an important prognostic factor of tumor behavior, as shown in the current study. Therefore, the 8th edition of UICC/AJCC staging system for cholangiocarcinoma has re-inserted tumor size into the TNM system again.

Cancer spreading through the lymphatic system is a common characteristic of cholangiocarcinoma, which is different from primary hepatocellular carcinoma that is rarely associated with lymph node metastasis. Hence, lymphadenectomy during the resection of ICC is highly recommended by most reports, despite no sufficient data supporting the true benefit of prophylactic lymphadenectomy [19]. This study also confirmed that lymph node involvement was a prognostic factor for cancer recurrence in patients after curative resection of ICC, indicating that lymphadenectomy might potentially provide benefit for these patients. Interestingly, the study also showed that vascular invasion was an independent prognostic factor for RFS of ICC. Vascular invasion is always a crucial prognostic factor for primary hepatocellular carcinoma after hepatic resection [20, 21]. However, vascular invasion is rarely reported as a prognostic factor for ICC after curative resection. To our knowledge, our current study might be the few to identify vascular invasion as a prognostic factor of ICC. Although this study might be limited by a relatively small number of patients in a single institute, we believe this observation to be valid. Additionally, further researches involving basic science and a larger number of patients should be conducted to confirm the significance of our results.

Although the study evaluated patients treated over four decades, the concept in terms of treatment strategies and surgical resection for ICC has not markedly changed during this period. As the study had analyzed patient outcomes based on different timeframes, the results showed that no significance was observed along with the time period at least in the institute. As such, early diagnosis accompanied by surgical resection is the gold standard for providing long term survival. Nonetheless, the majority of patients with ICC was initially asymptomatic or with vague symptoms that lead to late detection of malignancy and few patients eligible for curative surgical resection at early cancer stage. Although numerous risk factors were identified possibly association with the development of cholangiocarcinoma, none of the risk factors is specific to the disease. Currently, consensus on the implementation of risk stratification for disease surveillance is still unsettled despite current advancement of diagnostic tools. Therefore, the general outcome of patients with ICC remains not optimistic.

However, the high incidence of postoperative recurrence as this study is a major concern influencing the overall outcome of patients with ICC after surgical resection. Although postoperative adjuvant chemotherapy might be beneficial for patients following surgical resection, there is no consensus of adjuvant treatment strategies in terms of chemotherapeutic protocol and regimens to diminish the risk of postoperative recurrence nowadays [22]. Despite not being an independent prognostic factor, patients who received adjuvant chemotherapy after liver resection had a shorter disease free interval than those without adjuvant chemotherapy in the univariate analysis of the study. The theoretical explanation of this phenomenon could possibly be related to patient selection, in which patients who were subjected to chemotherapy had a considerable severe tumor status than other patients in the clinical practice. As a result, patients who had received adjuvant chemotherapy after liver resection had a relative poor outcome in terms of RFS. However, the present study was unable to clarify this issue, and further detailed analysis will need to confirm the validity of this observation.

Given the high incidence of recurrence after surgical resection, the management of postoperative recurrent cholangiocarcinoma has become more important. Although it remains arguable that the prognosis of patients who are suitable to undergo surgical resection for recurrent cancer is naturally better than that of patients who are unable to undergo surgical resection, an aggressive attitude in terms of surgical resection for postoperative recurrent cholangiocarcinoma still seems to be beneficial. As shown in this study, patients who had undergone repeat surgical resection for the recurrent disease would enjoy a better chance of survival. Nonetheless, for patients without surgical resection of postoperative recurrent cholangiocarcinoma, there is no doubt that palliative chemotherapy is better recommended. Palliative chemotherapy could also provide certain survival benefit for patients who are unable to receive surgical treatment for recurrent cholangiocarcinoma after adequate resection.

## Conclusion

The vague initial presentation of ICC may result in late detection at an advanced stage and lead to a low proportion of patients eligible for curative surgical resection. Meanwhile, the long-term incidence of postoperative cholangiocarcinoma recurrence is high, accounting for 74% of patients regardless of whether curative resection was performed. In line with previous studies, here the study identified many well-known prognostic factors that influence cancer recurrence after operation. Although patients with only intrahepatic recurrence had a better survival, the predictor of recurrent patterns after surgical resection was not identifiable based on the present study. Apart from that, the results suggest that an aggressive attitude in terms of surgical resection for postoperative recurrence might also be beneficial for the long-term outcome of a patient with ICC. Therefore, it is essential to regularly and frequently follow-up patients in the first few years after the operation to ensure early detection of recurrence at an operable stage. Eventually, in order to achieve better long-term outcomes for patients with ICC, the development of a treatment protocol that involves multidisciplinary modalities might be helpful in the future.

## Abbreviations

AJCC: American Joint Committee on Cancer
CT: Computed tomography
CUSA: Cavitron Ultrasonic Surgical Aspirator
ICC: Intrahepatic cholangiocarcinoma
MRI: Magnetic resonance imaging
OS: Overall survival
RFS: Recurrence-free survival
UICC: Union for International Cancer Control
